# Effective and safe use of immune checkpoint inhibitors for non‐small cell lung cancer in people living with HIV

**DOI:** 10.1111/hiv.70136

**Published:** 2025-11-05

**Authors:** Rebekka Mispelbaum, Tessa Hattenhauer, Christian Hoffmann, Clara Lehmann, Franz‐Georg Bauernfeind, Peter Brossart, Maximilian Christopeit, Winfried Alsdorf, Carsten Bokemeyer, Julian P. Layer, Julia Roider, Marcus Hentrich, Malte Benedikt Monin

**Affiliations:** ^1^ Department of Oncology, Hematology, Rheumatology and Immune‐Oncology University Hospital Bonn Bonn Germany; ^2^ Centre for Integrated Oncology (CIO), Aachen, Bonn, Cologne, Düsseldorf (ABCD) Aachen, partner‐site Bonn Germany; ^3^ Infektionsmedizinisches Centrum Hamburg Hamburg Germany; ^4^ Department I of Internal Medicine, Division of Infectious Diseases, Medical Faculty and University Hospital Cologne University of Cologne Cologne Germany; ^5^ German Center for Infection Research (DZIF) Bonn‐Cologne Germany; ^6^ Department of Oncology, Haematology and Bone Marrow Transplantation with Section Pneumology University Medical Center Hamburg‐Eppendorf Hamburg Germany; ^7^ Department of Radiation Oncology University Hospital Bonn Bonn Germany; ^8^ Institute of Experimental Oncology University Hospital Bonn Bonn Germany; ^9^ Department of Infectious Diseases University Hospital, Ludwig‐Maximilians‐Universität München Munich Germany; ^10^ Department of Medicine IV University Hospital, Ludwig‐Maximilians‐Universität München Munich Germany; ^11^ German Center for Infection Research (DZIF), Partner Site Munich Munich Germany; ^12^ Department of Internal Medicine III, Red Cross Hospital Munich Munich Germany; ^13^ Department of Internal Medicine I University Hospital Bonn Munich Germany

**Keywords:** immune checkpoint inhibition, non‐small cellular lung cancer, NSCLC, shock and kill

## Abstract

**Objectives:**

With the near normalization of life expectancy in people living with HIV through antiretroviral therapy, the management of age‐related comorbidities has become increasingly important. Non‐AIDS‐defining cancers now contribute significantly to both morbidity and mortality in this population, with non‐small cell lung cancer (NSCLC) being one of the leading causes of cancer‐related deaths among people living with HIV.

**Methods:**

Immune checkpoint inhibitors (ICIs) have revolutionized the treatment of NSCLC in the general population. However, people living with HIV have been largely excluded from pivotal clinical trials, resulting in limited evidence regarding safety and efficacy in this population. This review summarizes the main available literature on ICI therapy in people living with HIV with NSCLC.

**Results:**

The presented data from retrospective analyses and cohort studies suggest that people living with HIV benefit from ICI therapy, with similar response rates and survival outcomes as people living without HIV. The risk of immune‐related adverse events in people living with HIV was reported to be comparable to people living without HIV. Importantly, no significant effects of ICI on HIV viral load or CD4^+^ T cell count were reported.

**Conclusions:**

ICI therapy appears to be both safe and effective in people living with HIV with NSCLC. Optimal management of this patient population requires close interdisciplinary collaboration between oncologists and HIV care specialists. To enhance our understanding, broader inclusion of people living with HIV in clinical trials and the conduct of dedicated HIV‐specific studies would be essential.

## BACKGROUND

**FIGURE 1:**
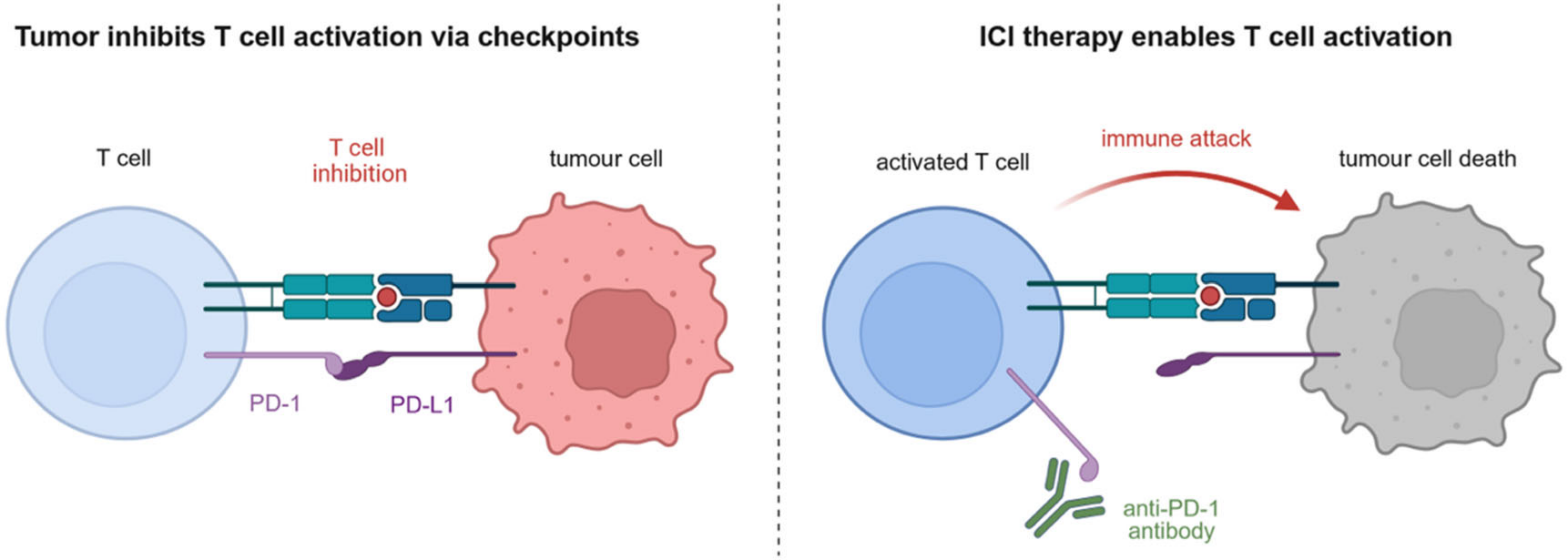
Mechanism of action of immune checkpoint inhibitors. Checkpoints, such as PD‐1, are blocked by immune checkpoint inhibitors, enhancing the activation and cytotoxic function of T cells with anti‐tumour effect (CD8^+^ cytotoxic T cells and CD4^+^ T helper cells). Created with BioRender.com. ICI, immune checkpoint inhibitors; PD‐1, Programmed Cell Death Protein 1; PD‐L1, Programmed Death‐Ligand 1.

Lung cancer is the second most frequently diagnosed cancer entity in men and the third in women, remaining the leading cause of cancer‐related death in men and the second leading cause in women across Europe [1]. In the last decade, the medical treatment landscape of non‐small cell lung cancer (NSCLC) has changed fundamentally. Immune checkpoint inhibitors (ICIs) have been established as an integral part of first‐line therapy for NSCLCs without targetable driver mutations. Checkpoints, such as PD‐1 (Programmed Cell Death Protein 1) and CTLA‐4 (Cytotoxic T‐Lymphocyte Associated Protein 4), are regulatory elements controlling the activation of the immune system and maintaining immune homeostasis. ICIs enhance the activation and cytotoxic function of T cells, which play a key role in anti‐tumour immunity, by blocking these checkpoints [2]. The anti‐tumour effect of ICIs primarily relies on reactivating exhausted CD8^+^ cytotoxic T cells and boosting the functions of CD4^+^ T helper cells [3, 4] (Figure 1).

The human immunodeficiency virus (HIV) targets immune cells via the CD4 receptor, affecting macrophages, monocytes and particularly CD4^+^ T cells (T helper cells). Untreated HIV infections lead to a significant decline in CD4^+^ T cells. However, modern antiretroviral therapy (ART) effectively suppresses viral replication, restores CD4^+^ T cell counts and reconstitutes immune competence, even in advanced disease [5]. In Europe, approximately 91% of all people living with HIV are aware of their HIV status, 93% of people who know their status are on ART and 92% of those on ART are virally suppressed and show an effective immune reconstitution, reflected by an increase in CD4^+^ T cell counts and, more importantly, by the recovery of functional immune competence [6]. As a result, people living with HIV have a near‐normal life expectancy if they adhere regularly and consistently to modern ART [7]. Epidemiological studies reflected that people living with HIV remain at increased risk of cancers and other comorbidities despite high CD4^+^ T cell counts, with near‐normal life expectancy observed mainly in men having sex with men with controlled HIV viraemia and CD4^+^ T cell counts >500 cells/μL when treated from 2015 onwards [8]. A proportion of people living with HIV, however, fail to fully restore CD4^+^ T cell functions or counts, leaving residual immunodeficiency that contributes to non‐AIDS events [9]. Overall, age‐related comorbidities such as cardiovascular events and malignant tumours are gaining increasing attention. By 2030, it is estimated that 17% of people living with HIV will develop a malignancy [10].

Since the introduction of ART, the incidence and disease burden of AIDS‐defining cancers (ADCs) have declined. In contrast, non‐AIDS‐defining cancers (NADCs) have shown a rising trend in both morbidity and mortality [11]. The degree of immunosuppression and disease severity at the time of HIV diagnosis, as indicated by low nadir CD4^+^ T cell counts, low CD4:CD8 T cell ratios, extended periods of immunodeficiency and a prior history of AIDS‐defining illness, has been reported to be associated with an increased incidence of NADCs [12, 13, 14, 15]. Furthermore, prolonged HIV viraemia, persistent immune dysregulation despite effective ART and HIV‐associated metabolic dysregulations were linked to an increased risk for NADCs [16, 17]. Non‐HIV‐related aspects, including ageing of the people living with HIV population, oncogenic viral co‐infections (EBV, HPV, HBV, HCV) and elevated rates of smoking, substance use and medication exposure, are other relevant factors [12, 18, 19, 20, 21].

People living with HIV have been reported to be at a higher risk of developing lung cancer compared to the general population [22]. Lung cancer is now the second most common cancer entity and the leading cause of cancer‐related deaths in people living with HIV [11, 23]. The elevated risk is largely driven by the disproportionately high prevalence of cigarette smoking in people living with HIV, which remains markedly higher than in HIV‐negative individuals across many regions [18, 24]. A retrospective analysis from southern Africa also revealed that people living with HIV were younger at the time of NSCLC diagnosis compared to people living without HIV and were less likely to be diagnosed at a limited stage [25]. Taking the current smoking patterns of people living with HIV in the US into account, up to 9.3% of people living with HIV aged 20–64 years are at risk of lung cancer‐related death. Even after adjusting for smoking status, a 1.7‐ to 2.4‐fold increased risk for lung cancer among people living with HIV remains, suggesting additional contributions from HIV‐related immune impairment caused by persistent CD4+ T cell deficiency, chronic inflammation and other comorbid factors as stated above [26, 27, 28].

However, HIV infection remains a common exclusion criterion in clinical studies, including the majority of trials that led to the approval of ICIs in lung cancer. Thus, data on the use of ICIs in NSCLC treatment in people living with HIV are limited. Sub‐analyses of phase III trials including patients with controlled HIV status are not reported [29]. Data from preclinical and singular clinical studies support safety and increase of HIV‐specific effector functions mediated by ICI therapy in people living with HIV [30, 31, 32]. Future studies should not only investigate the efficacy of ICIs in treating NSCLC among people living with HIV but also assess their potential effects on HIV replication and CD4^+^ T cell counts to better evaluate the safety of this treatment in this special population. Recent preliminary findings indicate that the tumour microenvironment of people living with HIV is altered and characterized by impaired anti‐tumour immune responses, which may influence the effectiveness of ICI and warrant further investigation [33].

## RESULTS OF THE LITERATURE REVIEW

A literature search was conducted in PubMed for articles published between January 2015 and September 2025. The following search terms were entered into PubMed: “Lung cancer AND HIV AND Immunotherapy”, “Lung cancer AND HIV AND checkpoint inhibitor”, “NSCLC AND HIV AND Immunotherapy”, “NSCLC AND HIV AND checkpoint inhibitor”, “NSCLC AND HIV AND atezolizumab”, “NSCLC AND HIV AND durvalumab”, “NSCLC AND HIV AND nivolumab”, “HIV AND NSCLC AND cemiplimab”, “HIV AND NSCLC AND pembrolizumab”, “HIV AND NSCLC AND ipilimumab”.

Clinical prospective studies, retrospective studies and registry studies investigating the use of ICI in people living with HIV with NSCLC were included, while case reports were excluded from further analysis. A total of seven relevant studies were identified. In a second step, studies including fewer than 15 patients were excluded, resulting in four studies remaining for the final analysis. Another criterion was a minimum follow‐up period of nine months.

This review aims to summarize the available evidence on the use of ICIs in people living with HIV with NSCLC and to address the existing knowledge gap in this field. The results of the included studies are summarized in Table 1 and discussed below. Currently, no data are available on the perioperative use of ICIs in NSCLC patients with HIV infection. Due to the lack of available data on consolidation therapy following radiochemotherapy, the only available case report is briefly discussed for this setting.

**TABLE 1:** Overview of Studies on people living with HIV and NSCLC.

Study design				Therapy response			Toxicity
Study (lung cancer cohort)	Therapy	HIV‐VL [copies/ml]/CD4^+^ T helper cells [cells/μl]	Follow‐up [months]	Tumour control	Median PFS [months] (95% CI)	Median OS [months] (95% CI)	AE total (grade ≥3)
CATCH‐IT [27]							
Retrospective analysis	ICI monotherapy: 56% (people living with HIV) versus 55% (people living without HIV)	N/A	14.8^a^	ORR: 28% (people living with HIV) versus 36% (people living without HIV) (*p* = 0.31)	2‐year PFS: 17.8% (people living with HIV) versus 18.4% (people living without HIV)	2‐year OS: 42.3% (people living with HIV) versus 41.5% (people living without HIV)	irAEs: 20.0% (12.0%) versus 22.0% (9.1%)
Matched NSCLC cohort: people living with HIV (*n* = 61) versus people living without HIV (*n* = 110)	ICI + chemotherapy: 44% versus 45%						
	1. therapy line: 61% versus 63%						
IFTC‐1602 CHIVA2 [28]							
Prospective phase II study people living with HIV with NSCLC + HIV‐VL <200 copies/ml (*n* = 16)	Nivolumab	HIV‐VL: 25 copies/ml (0–44)	23.6	DCR: 62.5%	3.4	10.9	Treatment‐related AEs: 75.0% (6.0%)
	2. or 3. therapy line	CD4^+^ T helper cells: 385/μl (187–778)					
French CANCERVIH registry study [29]							
Retrospective analysis (National CANCER VIH) people living with HIV with NSCLC (*n* = 21), HNSCC (*n* = 1), malignant melanoma (*n* = 1)	Nivolumab or pembrolizumab	Undetectable: 21/23 (91.3%)	NSCLC: 10.8	NSCLC: DCR: 9 (42.9%)	N/A	NSCLC: 10.7 (IQR: 8.4–15.4)	irAEs: 26.0% (8.7%)^b^
	1. (13%), 2. (55%) or ≥3. therapy line (36%)	<40 copies/ml: 1/23 (4.3%) 1/23 (unkown)		PR: 4 (19.0%) SD: 5 (23.8%)			
		CD4^+^ T helper cells: 364/μl					
OncoVIHAC ANRS CO24 cohort study [30]							
Prospective analysis people living with HIV with lung cancer (*n* = 65)	Durvalumab, atezolizumab, nivolumab, pembrolizumab ± chemotherapy	HIV‐VL: ≥50 copies/ml: 8/59 (13.6%) <50 copies/ml: 51/59 (86.4%)	9.2^c^	N/A	Estimate of PFS 18 months: 25.5%	Estimate of OS 18 months: 36.4%	irAEs: N/A (15.4%)
		CD4^+^ T helper cells: 374/μl (251–608)					

Abbreviations: AE, adverse event; DCR, disease control rate; HIV‐VL, HIV viral load; ICI, immune checkpoint inhibitor; irAE, immune‐related adverse event; N/A, not applicable; NSCLC, non‐small cell lung cancer; ORR, objective response rate; OS, overall survival; PFS, progression‐free survival; PR, partial remission; SD, stable disease.

^a^ Refers to the cohort of people living with HIV and NSCLC (*n* = 111).

^b^ Refers to the total cohort of cancer patients, including NSCLC (*n* = 23).

^c^ Refers to the total cohort of cancer patients, including NSCLC (*n* = 140).

### ICI therapy for advanced and metastatic NSCLC in people living with HIV

The CATCH‐IT consortium (Cancer Therapy Using Checkpoint Inhibitors in People Living With HIV‐International) retrospectively compared the use of ICIs in 390 people living with HIV with cancer, including 111 people living with HIV with NSCLC in the first‐line or recurrent setting [34]. For 61 people living with HIV diagnosed with metastatic NSCLC, a matched historical control group of people living without HIV with metastatic NSCLC was identified. Matching was performed based on age groups, sex, class of ICI, use of chemotherapy and number of lines of systemic therapy before ICI initiation. The distribution of patients with positive tumor proportion score was not balanced between the matched cohorts, and information on PD‐L1 status was not available for all patients. Patients were eligible for inclusion if they had received at least one dose of an ICI. No differences in overall response rates (ORR), progression‐free survival (PFS) and overall survival (OS) were observed between people living with HIV and people living without HIV with NSCLC. 22% of the people living with HIV group and 20% in the control group had an immune‐related adverse event (irAE), indicating no increased incidence associated with the HIV status. Analysis of the overall cohort, including various cancer types, showed no significant difference in the 24‐week cumulative incidence rate of irAE between patients with CD4^+^ T cell counts <200 cells/μL and those with ≥200 cells/μL. However, in patients with a baseline CD4:CD8 T cell ratio <0.4 versus ≥0.4, the irAE rate was significantly lower (10% vs. 26%; *p* = 0.01). For the six patients with an opportunistic infection at the time of ICI treatment initiation, no worsening of the infection was documented [34].

Nivolumab monotherapy as second‐ or third‐line therapy in people living with HIV with pretreated, advanced NSCLC (*n* = 16) was investigated in the non‐randomized phase II IFCT‐1602 CHIVA2 trial [35]. All patients had received at least one cycle of platinum‐based doublet chemotherapy before immunotherapy. Only people living with HIV treated with ART and with suppressed HIV‐RNA (<200 copies/mL) were eligible for inclusion, irrespective of the CD4^+^ T cell count. Most patients presented with adenocarcinoma and with negative tumour PD‐L1 expression. The disease control rate at eight weeks was 62.5%. Five patients showed progressive disease. Median PFS and OS were reported to be 3.4 months and 10.9 months, respectively. In 75.0% of patients, treatment‐related adverse events were observed but were mainly mild to moderate. During treatment, HIV‐RNA as well as CD4^+^ and CD8^+^ T cell counts remained stable, and no increased incidence of opportunistic infections was observed [35].

A retrospective sub‐analysis of the prospective French CANCERVIH registry of national multidisciplinary board discussions analysed cases of 575 people living with HIV with cancer [36]. Of 23 patients receiving immunotherapy with either pembrolizumab or nivolumab, 91.3% (*n* = 21) had NSCLC. The majority of patients (55%) received an ICI as second‐line treatment and were treated predominantly with nivolumab monotherapy. At ICI treatment initiation, all participants had an HIV‐RNA <40 copies/mL and the median CD4^+^ T cell count was 364/μl. Among patients with NSCLC, the disease control rate was 42.9% and the median survival was 10.7 months. ICI‐related adverse events occurred in 26.0% of the overall cohort, with two grade 3 irAEs. Among 10 patients with follow‐up data on CD4^+^ T cell counts, no clinically relevant change in the CD4^+^ T cell count was documented. One participant developed a detectable HIV‐RNA, attributed to ART interruption. In all other participants, HIV remained suppressed under ICI treatment [36].

The prospective French observational OncoVIHAC ANRS CO24 cohort study examined immune‐monotherapies and combined immune‐chemotherapies in people living with HIV with cancer [37]. Of 140 participants, 65 (46.0%) had lung cancer. The percentage of patients with NSCLC was not specified. Patients were eligible for inclusion regardless of CD4^+^ T cell count or viral load. The median follow‐up for the entire study cohort was 9.2 months. Estimated 18‐month OS among lung cancer patients was 36.4% and the estimated 18‐month PFS was 25.5%. irAE ≥ grade 3 occurred in 15.4% (10/65) of lung cancer patients with an incidence rate of 37.6 per 100 person‐years. The probability of experiencing at least one ≥ grade 3 irAE at 12 months was 16.6%. Based on a multivariable analysis, an increased risk of ≥ grade 3 irAE was more frequently observed in cancer patients with a low CD4^+^ T cell count (<200 cells/μL; incidence rate ratio (IRR): = 4.39, *p* < 0.0001) and positive cytomegalovirus serology (IRR = 2.76, *p* = 0.034). For 81 patients from the overall study population, a median of 3 HIV‐RNA measurements was obtained during the study period. Among 67 patients with undetectable viral load (<50 copies/mL) at ICI initiation, 92.5% (62/67) remained suppressed at last follow‐up. The CD4^+^ and CD8^+^ T cell counts, as well as the CD4:CD8 T cell ratio, remained stable across the cohort [37].

### Consolidation therapy with ICI following chemoradiation in people living with HIV

The PACIFIC trial in people living without HIV with stage IIIA/B NSCLC demonstrated significant improvements in PFS and OS with durvalumab consolidation following definitive chemoradiation. The 5‐year OS rate was 42.9% in the durvalumab arm versus 33.4% in the placebo group. Based on these findings, durvalumab was approved for adult patients with locally advanced, unresectable NSCLC whose tumours express PD‐L1 ≥ 1% and who have not progressed after platinum‐based chemoradiotherapy. Data for ICI consolidation therapy in people living with HIV are limited to a single case report of a 45‐year‐old HIV‐positive male, diagnosed with stage IIIB lung adenocarcinoma. He received concurrent chemoradiotherapy, resulting in tumour shrinkage. Durvalumab consolidation was discontinued after 4 months due to disease progression. No irAE or safety concerns occurred during ICI treatment [38].

## DISCUSSION

ICI therapy has become an essential part of oncological treatment for patients with NSCLC. Due to the immune‐modulatory nature of ICI therapy, concerns about the safety and efficacy of ICI treatment in people living with HIV must be considered. Data on the use of ICI for NSCLC in people living with HIV remain limited. Many phase II trials for immunotherapy either excluded people living with HIV or did not provide subgroup analyses specific to this population (EMPOWER‐Lung 1, CheckMate 817) [35]. Therefore, the available data are primarily limited to retrospective analyses, which lack homogeneity and adequate patient numbers (Table 1).

The authors of the available studies investigating ICI treatment in people living with HIV with NSCLC conclude that there is currently no evidence to suggest a reduced efficacy of ICI therapy in this population. Spano et al. reported an OS of 10.7 months in people living with HIV with NSCLC treated with ICI therapy (above all second‐line treatment) [27]. This was considered comparable to the results of the phase III trials CheckMate 017 and CheckMate 057, which evaluated Nivolumab as second‐line treatment and reported OS rates of 9.2 and 12.2 months, respectively [39, 40]. In addition, a matched cohort study of people living with HIV with NSCLC treated with ICIs showed comparable efficacy outcomes to people living without HIV [25]. After adjusting for key clinical factors, ORRs were similar between the two groups [34].

The main complication of ICI therapies is irAEs, which may present as colitis, pneumonitis, hepatitis, immune‐mediated skin reactions or other organ toxicities. These adverse events can potentially be fatal and are therefore of critical importance in the safety assessment of ICI therapy. The OncoVIHAC ANRS CO24 cohort study was developed to assess the real‐world safety of ICI in people living with HIV with cancer, predominantly including lung cancer patients [37]. The rate of irAEs during the observation period was deemed comparable to the historical general population, and most irAEs were reversible by steroid treatment [37]. Data on the incidence of severe irAEs in people living with HIV treated with ICIs are consistent with the rates observed in people living without HIV [34]. This is supported not only by the presented studies, focusing on NSCLC patients, but also by other research involving people living with HIV with various types of cancer treated with ICIs [32, 36, 41, 42, 43, 44]. Moreover, in trials that included patients receiving chemoimmunotherapy, the addition of chemotherapy was not reported to have unexpected toxicities in people living with HIV [34, 37].

The OncoVIHAC ANRS CO24 cohort study also investigated risk factors for the incidence of ≥ grade 3 irAEs in people living with HIV [37]. Based on a multivariable analysis, an increased risk of ≥ grade 3 irAE was demonstrated for patients with a low CD4^+^ T cell count (<200 cells/μL) [37]. In contrast, in the CATCH‐IT study, no correlation between lower CD4^+^ T cell counts and cumulative incidence rates of any grade irAE was identified [34]. However, for patients with a CD4:CD8 T cell ratio <0,4, a significantly lower risk for irAE was reported [34]. The influence of CD4^+^ T cell counts on the risk of irAE in people living with HIV remains complex. On the one hand, theoretical considerations suggest that a low CD4^+^ T cell count may be associated with chronic immune dysfunction, potentially increasing the risk of immune‐related toxicity from ICI [37]. On the other hand, higher CD4^+^ T cell counts may be linked to an increased risk of irAE due to a better functional immune system showing a stronger immune response to ICI therapy [45].

In addition, safety data for people living with HIV with incomplete immune reconstitution, who are suffering from opportunistic infections, are limited. The six reported cases of opportunistic infections at initiation of ICI treatment in the retrospective CTACH‐IT did not show any worsening of the infection [34]. Treatment decisions must be made individually for such patients, guided by the urgency of cancer therapy.

Besides safety concerns related to irAEs, the potential impact of ICI therapy on HIV parameters is essential to consider. Our review revealed no clinically relevant impact on plasma HIV‐RNA viral load during ICI treatment [34, 35, 36]. These findings are consistent with data from studies in various cancer entities, where HIV‐RNA remained stable in over 90% of participants receiving ICI therapy [46]. Increases in HIV viraemia were infrequent (5.8%), low‐level (mean 1.03 log₁₀ copies/ml), transient and clinically irrelevant, with no need for modification of antiretroviral treatment or ICI therapy [46]. Furthermore, no clinically meaningful impact on CD4^+^ T cell counts was observed in the presented studies on ICI treatment in people living with HIV with lung cancer [34, 35, 36, 37]. In the systematic review by Abbar et al. addressing the safety of ICI therapy in people living with HIV, CD4^+^ T cell counts were reported as stable in 60.7%, increased in 24.6% (median + 171/μL) and substantially decreased in 14.7% (median − 200/μL) of participants. However, the authors conclude that these changes have no significant impact on clinical practice [46].

**FIGURE 2:**
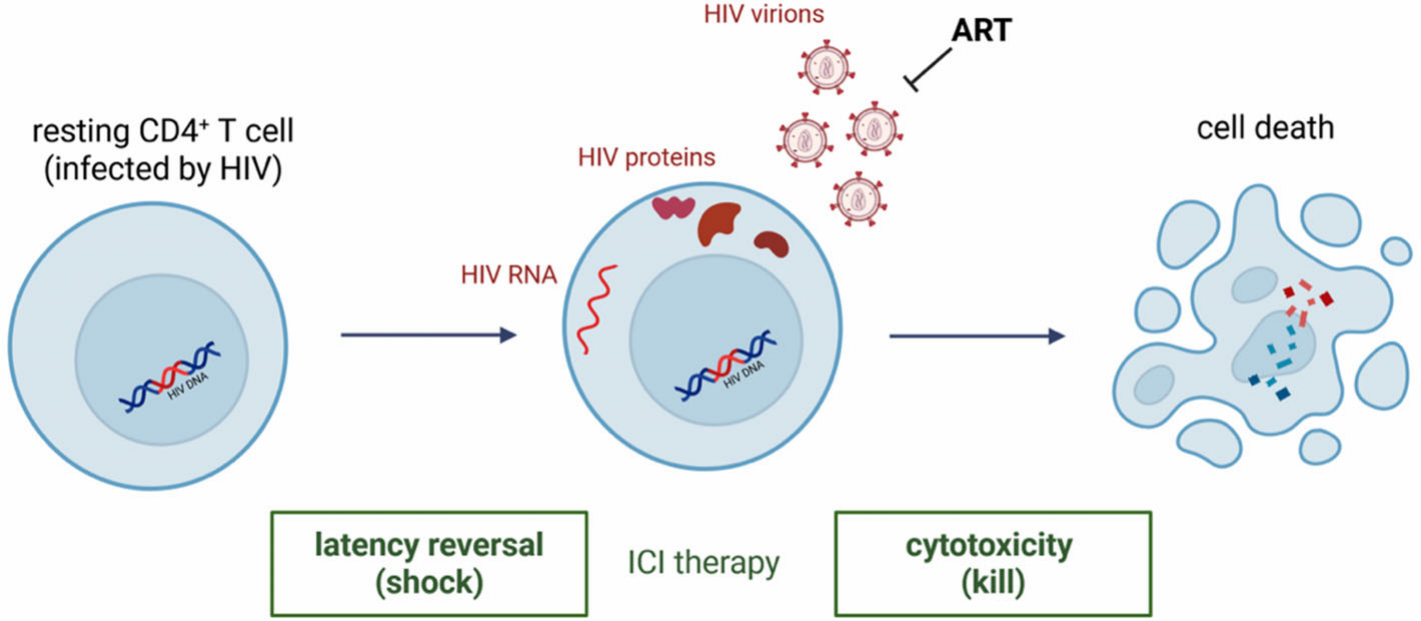
Shock and kill approach. Immune checkpoint inhibitors may reactivate latent HIV reservoirs in people living with HIV under effective ART (shock) and subsequently eliminate infected cells via enhanced HIV‐specific immune responses (kill). Created with BioRender.com; modified from [38]. CPI, Checkpoint inhibitor.

Besides oncological indications, the use of ICIs is discussed as a potential HIV cure strategy, addressing both the “shock and kill” approach (Figure 2) [47] and restoring effector functions of HIV‐specific T cells. This concept aims to reactivate latent HIV reservoirs in people living with HIV under effective ART (shock) and subsequently eliminate infected cells via enhanced HIV‐specific immune responses (kill). The HIV reservoir consists of latently infected CD4^+^ T cells overexpressing immune checkpoints and harbouring integrated, replication‐competent HIV DNA, which persist despite effective ART [48, 49]. ICIs may contribute to both steps by reactivating latently HIV‐infected CD4^+^ T cells (shock) and enhancing cytotoxic CD8^+^ T cell responses (kill). However, corresponding immunological data are reported in only about 10% of published cases on ICI use in people living with HIV [50, 51, 52]. For the time being, the isolated effect of ICIs on the HIV reservoir appears too limited, indicating that ICIs will likely need to be combined with other synergistic interventions in future cure‐directed clinical trials [46]. Nevertheless, this concept should be interpreted as supporting—rather than discouraging—the use of ICIs in people living with HIV, particularly when clinically indicated for oncological reasons.

Despite data suggesting similar frequencies of adverse events and efficacy, consistent with cancer studies and the use of ICI therapies in people living without HIV, several limitations should be noted. Most evidence is derived from retrospective and non‐randomized studies, and the overall number of patients remains small. Prospective studies in people living with HIV are challenging but warranted also addressing HIV‐related parameters. At a minimum, HIV infection per se should not be an exclusion criterion, and people living with HIV with suppressed viral load and immune reconstitution (CD4^+^ T cell count >200/μL) could reasonably be included in clinical and/or pivotal trials. A notable example is the prospective CheckMate 817 study in patients with metastatic NSCLC, which included four people living with HIV with suppressed viral load and adequate immune reconstitution (CD4^+^ T cell count >200/μL) [53]. In this study, flat‐dose nivolumab plus weight‐based ipilimumab demonstrated manageable safety and durable efficacy, consistent with outcomes in people living without HIV supporting the inclusion of well‐controlled people living with HIV in clinical trials and supporting the use of ICIs in this population [53].

## CONCLUSION

The available data suggest that immune checkpoint inhibitors are safe and effective in people living with HIV with NSCLC, supporting their virological and immunological safety in this population. However, the number of patients included in current studies is limited, and data on dual checkpoint blockade are lacking. Close interdisciplinary collaboration between oncology and HIV care providers is essential—particularly regarding ART management, potential drug interactions and regular monitoring of immune status and HIV viral load.

## CONFLICT OF INTEREST STATEMENT

R.M. received travel fees and/or honoraria from Takeda, Janssen‐Cilag and Johnson & Johnson. T.H. received travel fees and/or honoraria from Takeda, Janssen‐Cilag, Johnson & Johnson and Regeneron Pharmaceuticals. C.H. received travel fees and/or honoraria from Gilead Sciences, Janssen‐Cilag, MSD, Recordati and ViiV Healthcare. CL received honoraria for lectures or consultations from Abbvie, BMS, MSD, Novartis, Gilead, GSK, Janssen, Pfizer and ViiV; travel support from Gilead. F.‐G.B. declares no conflicts of interest. P.B. declares no conflicts of interest. M.C. received honoraria from BeiGene, JAZZ, GSK, Roche, Tillotts, AstraZeneca, AbbVie, Janssen‐Cilag, Ciba Geigy, Boehringer Ingelheim, Pfizer, MSD, Takeda, Medac, Servier and BMS. He has a US Patent (PCT/US2014/72474) ‘EPIGENETIC STEM CELL COMMITMENT ASSOCIATED SIGNATURE’, shared with Ulrich Steidl, Boris Bartholdy and Amit Verma. W.A. received travel fees and/or honoraria from BioNTech, Immatics, Johnson & Johnson, GSK, Astellas, AstraZeneca and clinical advisory board membership from Johnson & Johnson as well as research funding (received by institution) by Affimed and BioNTech. C.B. received honoraria from AOK Germany, Astra Zeneca, Bayer Healthcare, BioNTech, Eurobio, Lindis Biotech, med update, Merck Serono, Oncology Drug Consult CRO and Roche Pharma, Invited Speaker, Personal. J.P.L. reports stocks and travel expenses from TME Pharma AG, travel expenses and honoraria from Carl Zeiss Meditec AG, honoraria and clinical advisory board membership from OncoMAGNETx Inc., stocks and honoraria from Siemens Healthineers AG and stocks from Bayer AG and BioNTech AG. J.R. declares no conflicts of interest. M.H. received travel fees and/or honoraria from Amgen, Beigene, Gilead, Janssen, Jazz Pharma, Menarini, Merck, Recordati and Sanofi. M.B.M. received travel fees and/or honoraria from AbbVie, AstraZeneca, Gilead Sciences, Janssen‐Cilag, Johnson & Johnson, Novavax, Pfizer, Takeda and Virology Education. However, these activities have no potential conflicts of interest with the content of the manuscript.

## Data Availability

The data that support the findings of this study are available on request from the corresponding author. The data are not publicly available due to privacy or ethical restrictions.
